# Phase I first-in-human study of TAK-285, a novel investigational dual HER2/EGFR inhibitor, in cancer patients

**DOI:** 10.1038/bjc.2011.590

**Published:** 2012-01-12

**Authors:** T Doi, H Takiuchi, A Ohtsu, N Fuse, M Goto, M Yoshida, N Dote, Y Kuze, F Jinno, M Fujimoto, T Takubo, N Nakayama, R Tsutsumi

**Affiliations:** 1 Division of Gastrointestinal Oncology, National Cancer Center Hospital East, 6-5-1 Kashiwanoha, Kashiwa, Chiba 277-8577, Japan; 2 Cancer Chemotherapy Center, Osaka Medical College Hospital, 2-7 Daigakucho, Takatsuki, Osaka 569-8282, Japan; 3 Pharmaceutical Research Division, Takeda Pharmaceutical Company Limited, 2-17-85 Jusohonmachi, Yodogawa-ku, Osaka 532-8686, Japan; 4 Clinical Data Science Division, Takeda Bio Development Center Limited, Sapia Tower, 1-7-12 Marunouchi, Chiyoda-ku, Tokyo 100-0005, Japan; 5 Preclinical and Clinical Pharmacology Division, Takeda Bio Development Center Limited, Sapia Tower, 1-7-12 Marunouchi, Chiyoda-ku, Tokyo 100-0005, Japan; 6 Clinical Development Division, Takeda Bio Development Center Limited, Sapia Tower, 1-7-12 Marunouchi, Chiyoda-ku, Tokyo 100-0005, Japan

**Keywords:** first-in-human, phase I TAK-285, epidermal growth factor receptor, dual erbB protein kinase inhibitor family, receptor tyrosine kinase inhibitor

## Abstract

**Background:**

This phase I first-in-human study was conducted in Japanese patients to investigate the safety, pharmacokinetics (PKs), and determine the maximum tolerated dose (MTD) of oral TAK-285, a novel dual erbB protein kinase inhibitor that specifically targets human epidermal growth factor receptor (EGFR) and HER2.

**Methods:**

The TAK-285 dose was escalated until MTD was determined. A second patient cohort received TAK-285 at the MTD for at least 4 weeks.

**Results:**

In all, 26 patients received TAK-285 at doses ranging from 50 to 400 mg once daily (q.d.) or twice daily (b.i.d.); 20 patients made up the dose escalation cohort and the remaining 6 patients were the repeated administration cohort. TAK-285 was well tolerated. Dose-limiting toxicities noted in two patients who received 400 mg b.i.d. were grade 3 increases in aminotransferases and grade 3 decreased appetite. Consequently, the MTD was determined to be 300 mg b.i.d. Absorption of TAK-285 was rapid after oral dosing, and plasma exposure at steady-state increased in a dose-proportional fashion for doses ranging from 50 to 300 mg b.i.d. A partial response was observed for one patient with parotid cancer who received 300 mg b.i.d.

**Conclusion:**

The toxicity profile and PK properties of oral TAK-285 warrant further evaluation.

Dimerisation of the human epidermal growth factor receptor (EGFR) protein family members, including HER1/EGFR and HER2, activates intracellular kinase and initiates a phosphorylation cascade that, in tumour cells, results in enhanced cellular proliferation and survival. Especially in the case of dimers that contain HER2, such activation of signal transmission can be persistent and potent, and under these circumstances is associated with high cellular differentiation and abnormal growth (Reid *et al*, 2007).

Clinically, HER2 and EGFR overexpression and the associated increase in cellular signal transduction is a common feature of tumours such as breast cancer and gastric cancer, and is associated with aggressive disease (Yonemura *et al*, 1991; Salomon *et al*, 1995; Nicolini *et al*, 2006). The prognosis is worse for such patients than for non-overexpressing patients. This also applies to many other cancer types such as colon cancer, ovarian cancer and bladder cancer, and small molecular weight chemotherapeutic agents or antibodies that target EGFR and HER2 and inhibit their activity have been proven to be clinically effective in overexpressing cancers (Hynes and Lane, 2005; Shepherd *et al*, 2005; Thatcher *et al*, 2005; Moore *et al*, 2007; Mok *et al*, 2009).

TAK-285 is a novel low-molecular weight compound that was designed and synthesised by Takeda Pharmaceutical Company, Osaka, Japan and has been shown to selectively and potently inhibit HER2 and EGFR kinase activities. Biochemically, TAK-285 inhibits HER2 and EGFR phosphorylation, with 50% inhibition concentrations of 17 and 23 nmol l^–1^, respectively (Aertgeerts *et al*, 2011).

The antitumour activity of TAK-285 was evaluated in several murine models employing HER2- or EGFR-overexpressing human tumour xenografts such as BT-474, 4-1 ST and A431. These studies revealed that orally administered TAK-285 effectively inhibited xenograft growth and this effect appeared to correlate with its ability to inhibit EGRF and HER2 (Iwahara *et al*, 2008). Additionally, in rodent and primate toxicity models, TAK-285 was well tolerated and induced toxicities observed with other compounds possessing a similar mechanism of action. TAK-285 also demonstrated potentially no exhibition of elevated cardiac risks whereas other tyrosine kinase inhibitors can elicit secondary effects including heart toxicity (Shell *et al*, 2008). In total, these non-clinical studies suggest that TAK-285 may possess exploitable antineoplastic activity and consequently a phase I first-in-human study in patients with solid tumours was conducted in Japan.

## Patients and methods

### Trial design

This was a phase I, multicentre, open-label study, conducted to investigate the safety, pharmacokinetics (PKs), and determine the maximum tolerated dose (MTD) of oral TAK-285 in patients with solid tumours. Two cohorts were planned for this study: a dose escalation cohort and a repeated administration cohort. In the dose escalation cohort, patients received a single oral dose of TAK-285, followed by 2–6 days of observation without treatment, followed by treatment with the same dose if the safety was confirmed. In this cohort, patients received TAK-285 once weekly. One cycle was 4 weeks, consisting of 3 weeks of treatment and 1 week of observation without treatment. TAK-285 was given once daily (q.d.) or twice daily (b.i.d.). The dose was escalated from a starting dose of 50 mg until the MTD was determined. In the repeated administration cohort, patients were treated with oral TAK-285 at the MTD for at least 4 weeks in order to confirm safety. Patients continued to be treated with TAK-285 at the same dose level if the treatment was well tolerated and there was no evidence of progressive disease (PD).

The study was conducted in accordance with the protocol approved by the institutional review boards of the participating institutions, and with the Harmonized Tripartite Guideline of the International Conference on Harmonization for Good Clinical Practice.

### Patient eligibility

Patients with histologically/cytologically confirmed metastatic or advanced cancer that was unresponsive to standard therapy were eligible for this study, provided that the following criteria were met: Eastern Cooperative Oncology Group performance status of 0–1; age of 20–74 years; life expectancy of at least 12 weeks; adequate bone marrow and organ function; at least 1 measurable lesion based on Response Evaluation Criteria in Solid Tumours (RECIST) (Therasse *et al*, 2000) (patients with no measurable lesion were acceptable for the dose escalation cohort only); and no previous therapy with an EGFR or HER2 inhibitor (except for trastuzumab).

### Dose escalation scheme

In the dose escalation cohort, the dose of TAK-285 was decided by consideration of adverse events (AEs) observed during the first cycle. If one of three patients had a dose-limiting toxicity (DLT), another three patients were added to the cohort. If none of three patients had a DLT, the dose in subsequent patients was increased to the next level. If there remained only one patient having a DLT, the dose was also increased to the next level, however, if >1 patient had a DLT, there was no progression to the next level. Dose escalation was continued until the MTD was determined. The dose of TAK-285 was increased by 100 or 40% in accordance with accelerated titration designs reported previously (Simon *et al*, 1997). A DLT was defined as any TAK-285-related grade 4 haematological toxicity, grade 3 or worse non-haematological toxicity, grade 3 or worse neutropenia (<1000 mm^–3^) with fever of 38 °C or higher, or toxicity resulting in cessation of treatment for >21 consecutive days (including the stipulated period of observation without treatment).

### Endpoints

The primary study endpoints were to determine the MTD as well as PK profiles of TAK-285 and its metabolite, M-I (data on file, Takeda Pharmaceutical Company Limited). The secondary endpoints were objective response rate (complete response (CR) and partial response (PR)), disease control rate (CR, PR and stable disease (SD), for at least 12 weeks), and time to tumour progression, defined as the time from the first dose of TAK-285 until disease progression or death. Tumour response was assessed every 4 weeks by RECIST version 1.0 (Therasse *et al*, 2000).

### Safety assessments

Safety evaluations included vital signs (oxygen saturation, body temperature, breathing rate, blood pressure and pulse), clinical laboratory tests, lung function tests (pulmonary surfactant protein-A, pulmonary surfactant protein-D, Krebs von den Lunge-6 and arterial blood gas analysis), chest X-ray, and 12-lead electrocardiogram (ECG). These tests were performed weekly with the exception of chest X-rays (every 4 weeks), arterial blood gas analysis (at screening) and ECG (4 time points each at screening, on day 1 and day 8, after DLT assessment, one time point every 4 weeks). All ECG charts were submitted to the ECG evaluation committee to assess cardiac function. Adverse events were graded based on the National Cancer Institute Common Toxicity Criteria for Adverse Events, version 3.0 (Bethesda, MD, USA).

### PK analyses

In the dose escalation cohort, plasma samples for PK analysis were collected at predose and up to 72 h after single dose administration and on day 21 after repeated administration of TAK-285. Urine samples were also collected up to 24-h postdose. In the repeated administration cohort, plasma samples for PK analysis were collected at predose and up to 12-h postdose on days 1 and 28. Urine samples were also collected up to 12-h postdose each day. Concentrations of TAK-285 and M-I in plasma and urine were determined using validated liquid chromatography tandem mass spectrometry (LC-MS/MS) methods. The plasma and urine samples were treated by liquid–liquid extraction and subjected to LC-MS/MS equipped with a reversed-phase column. The lower limit of quantification for both TAK-285 and M-I was 0.2 ng ml^–1^ in plasma and 2 ng ml^–1^ in urine when 50 *μ*l of the plasma sample and 55 *μ*l of the urine sample (containing 5 *μ*l of Tween 80 solution) were analysed. Tween 80 solution was added to the urine sample to prevent the adsorption of analytes onto the sample containers. The accuracy of the plasma assay (percentage deviation from nominal) ranged from −10.8 to 11.0% for TAK-285, and from −14.0 to 13.8% for M-I. The accuracy of the urine assay ranged from −7.7 to 7.0% for TAK-285 and from −9.5 to 8.8% for M-I. Pharmacokinetic parameters of TAK-285 and M-I were estimated for each patient using noncompartmental methods with Phoenix WinNonlin Version 6.1 (Pharsight, Mountain View, CA, USA).

### Pharmacodynamic analyses

At screening and on day 15 after the start of repeated administration, 20 ml of peripheral blood was obtained and divided into two 10 ml exclusive use containers (CellSave tubes) filled with 300 *μ*l of preservative solution (4.6% Na-ethylenediaminetetraacetate, 36% cell preservative, 0.36% polyethylene glycol and 0.4% inert ingredients). The samples were stored at room temperature and processed within 72 h after sampling. The CellSearch System (Veridex LLC, Raritan, NJ, USA) was used for the isolation and enumeration of circulating tumour cells (CTCs), which were defined as nucleated cells lacking cluster of differentiation 45 and expressing cytokeratin.

## Results

### Patient characteristics

A total of 26 patients were enrolled between July 2007 and May 2010, and received at least one dose of TAK-285. Demographic characteristics of patients are summarised in Table 1. Safety and efficacy were analysed for all 26 patients.

### DLT and MTD

The 26 patients received TAK-285 at a dose ranging from 50 to 400 mg q.d. or b.i.d., 20 patients made up the dose escalation cohort and the remaining 6 patients were the repeated administration cohort. Dose-limiting toxicities observed during the study were grade 3 increased alanine aminotransferase (grade 3) and increased aspartate aminotransferase in one patient, and decreased appetite in a second patient. Both patients were treated with TAK-285 400 mg b.i.d.; hence, the MTD was determined to be 300 mg b.i.d. In the repeated administration cohort, six patients received the MTD continually for 36–169 days.

### Safety

Adverse events were observed in all 26 patients who received TAK-285; 22 (84.6%) of these AEs were considered to be related to TAK-285 treatment.

The most common grade of AEs related to TAK-285 was grade 2 or lower in 17 patients (65.4%), grade 2 in 11 (42.3%), grade 3 in 5 (19.2%); there were no grade 4 AEs related to TAK-285 administration. As noted above, grade 3 AEs related to TAK-285 treatment included DLTs in two patients: increased alanine aminotransferase and increased aspartate aminotransferase in one patient, and decreased appetite in a second patient. These patients were withdrawn from the study because their DLTs resulted in permanent discontinuation of TAK-285 treatment. No other patients were withdrawn from the study because of TAK-285 treatment-related AEs. One of two patients with DLTs had myocardial ischaemia, which was the only serious AE related to TAK-285 treatment observed during the study. Two patients died during the study; in both cases, the cause of death was aggravation of the primary disease and was considered to be unrelated to TAK-285 treatment. The time period from last dose to death was 33 and 25 days, respectively.

Table 2 shows frequently reported AEs (having an overall incidence of ⩾10%). The most frequently reported AEs were increased alanine aminotransferase and aspartate aminotransferase, followed by rash, increased blood bilirubin and diarrhoea. The incidences of AEs related to TAK-285 treatment were similar to the incidences of AEs in general. A slight dose-dependent relationship was observed for these AEs. Grade 2 rash and diarrhoea were reported in the dose groups in which DLTs were observed, while only grade 1 AEs were observed in lower dose levels.

No clinically significant changes were observed in lung function tests, chest X-rays, vital signs or ECGs.

### Pharmacokinetic

**Figure 1:**
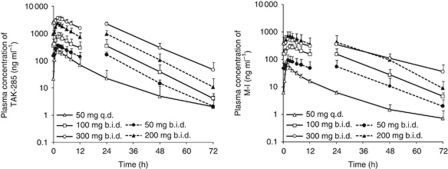
Mean plasma concentration-time profile of TAK-285 or M-I on day 21 in the dose escalation cohort. Mean±s.d. (*n*=3–4 at each dose level); TAK-285 was additionally dosed at 12 h postdose b.i.d.

**Figure 2:**
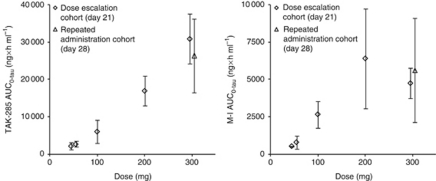
Relationship between dose and AUC_0−tau_ of TAK-285 or M-I after multiple oral administration of TAK-285 (day 21 or day 28). Data are mean±s.d. (*n*=3–6 at each dose level).

The PK parameters of TAK-285 and M-I are summarised in Tables 3 and 4, respectively. Plasma concentrations of TAK-285 and M-I reached the maximum (*C*_max_) within 2.5 h after single oral administration. The estimated terminal-phase half-life (*t*_1/2_) was 6–9 h for TAK-285 and 7–10 h for M-I at all doses. The time to reach the maximal plasma concentration (*T*_max_) and *t*_1/2_ were not remarkably changed after multiple dosing. On day 21, mean plasma concentrations of TAK-285 increased over a dose range of 50–300 mg b.i.d., and mean concentrations of M-I increased over the TAK-285 dose range of 50–200 mg b.i.d. (Figure 1). A dose-proportional increase in the area under the plasma concentration-time curve over the dosing interval (AUC_0−tau_) was suggested for TAK-285 after multiple dosing at doses ranging from 50 to 300 mg b.i.d. but was not clearly indicated for M-I (Figure 2). The accumulation ratios of *C*_max_ were 1.2–3.6 for TAK-285 and 1.2–2.6 for M-I. The accumulation ratios of AUC were 1.4–4.6 for TAK-285 and 1.4–3.7 for M-I. The mean cumulative excretion ratios of TAK-285 and M-I in urine (up to 12- or 24-h postdose following single or multiple dosing) were below 0.02% of the dose.

### Pharmacodynamics

Circulating tumour cell samples both at screening and on cycle 1 day 15 after the start of repeated administration were obtained and evaluated in all patients except for those who were removed from study by cycle 1 day 15 and 1 patient with a coagulated blood sample on cycle 1 day 15. Overall, seven patients had unfavourable baseline counts (⩾5 cells per 7.5 ml blood), none of which converted to a favourable count after TAK-285 treatment. Analysis of CTC data did not show significant changes after treatment with TAK-285.

### Antitumour activity

One patient with parotid cancer (3.8%) achieved a PR lasting for 56 days. However, the remaining patients were reported as PD.

The maximum percentage decrease from baseline in tumour size (sum of measured lesions) was evaluated separately for all patients. Only one patient in the dose escalation cohort showed reduction in tumour size, whereas three of six patients in the repeated administration cohort showed reduction in tumour size.

The objective response rate and disease control rate were each 3.8% (1 of 26 patients), and the median time to tumour progression was 58 days.

## Discussion

Clinically, TAK-285 was very well tolerated in spite of the fact that AEs were observed in all patients. The most frequent AEs were increased alanine aminotransferase and increased aspartate aminotransferase, followed by rash, increased blood bilirubin and diarrhoea; these were similar to AEs seen with other inhibitors of the EGFR family of tyrosine kinases (Hidalgo *et al*, 2001; Herbst *et al*, 2002; Ranson *et al*, 2002; Arora and Scholar, 2005; Lacouture *et al*, 2006). The incidences of frequently reported AEs appeared to correlate with dose; however, because of the limited number of patients studied this relationship could not be confirmed.

Dose-limiting toxicities were observed in two patients receiving 400 mg b.i.d. in the dose escalation cohort, but were not observed in the repeated administration cohort receiving the MTD. A serious AE, myocardial ischaemia, related to TAK-285 was reported in one of two patients with DLTs (receiving 400 mg b.i.d.). This event was considered to be related to TAK-285 treatment because diarrhoea that developed after the start of treatment with TAK-285 was suspected to have aggravated pre-existing ischemic heart disease. The remaining serious AEs were regarded as unrelated to TAK-285 treatment. Two patients died during the study, and the cause of death was considered to be unrelated to TAK-285 treatment for both patients.

It was interesting that pneumonitis was not reported in this study and is in contrast to findings seen with gefitinib, another EGFR tyrosine kinase inhibitor (Inoue *et al*, 2003). In addition, no significant changes in ECG were reported despite the known expression of HER2 in cardiac myocytes (Slamon *et al*, 2001; Seidman *et al*, 2002; Negro *et al*, 2004). Similarly, rash induced by TAK-285 was relatively mild (i.e., grade 2 or lower; grade 1 in a majority of patients), compared with that seen with gefitinib and erlotinib (Shepherd *et al*, 2005; Thatcher *et al*, 2005). The correlation between the incidence of diarrhoea and dose, which was previously reported with lapatinib (Burris *et al*, 2009), was also examined in our study; a similar although smaller correlation was observed with TAK-285. Diarrhoea was relatively mild (i.e., grade 2 or lower) in our study, and was grade 1 in a majority of patients.

In addition to evaluating the PK of TAK-285, the PK of the TAK-285 metabolite, M-I, was also evaluated. Laboratory studies revealed that M-I inhibits the kinase activities of HER2, EGFR, and HER4 with 50% inhibition concentrations of 98, 29 and 280 nmol l^–1^, respectively (data on file, Takeda Pharmaceutical Company Limited). The *C*_max_ of TAK-285 and M-I was observed up to 2.5 h after single dose administration at all doses, indicating that absorption of TAK-285 was relatively rapid after oral administration. The *t*_1/2_ values of M-I mirrored those of TAK-285 at all doses, and their concentrations in plasma declined in a similar manner. The accumulation of TAK-285 and M-I following multiple dose administration was considered to be moderate because the mean accumulation ratios for *C*_max_ and AUC were below 4.6 for both. A dose-proportional increase in exposure to TAK-285 at steady state was indicated over the dose range tested (50–100 mg b.i.d.), but the exposure to M-I did not increase with dose at 300 mg b.i.d. This suggests that metabolism of TAK-285 to M-I by hydroxylation may be saturated at higher doses. Urinary excretion of TAK-285 and M-I was negligible and indicated that renal excretion does not contribute significantly to the clearance of either TAK-285 or M-I.

The relationship between CTCs and prognosis has been reported for prostate and breast cancer (Cristofanilli *et al*, 2004; Danila *et al*, 2007), and the CTC test was approved by the FDA in January 2004. The association between CTCs and tumour response was not assessed sufficiently, because only one patient reported as PR, whose CTC number on day 15 was not available because of a coagulated blood sample. In this study, there were no significant changes in CTC number that might suggest therapeutic efficacy.

The antitumour response was rated as PR in one patient with parotid cancer in the repeated administration cohort. HER2 has been reported to be highly expressed in parotid cancer (Cornolti *et al*, 2007; Williams *et al*, 2010), and lapatinib was reported to be effective for the treatment of parotid cancer in a phase I study (Burris *et al*, 2009).

In summary, based upon its safety, tolerability profile, PK characteristics and potential antineoplastic activity in patients with advanced solid tumours, further evaluation of TAK-285 for the treatment of patients with solid tumours appears warranted.

## Figures and Tables

**Table 1:** Patient characteristics

	**Dose level of TAK-285 (mg)**							
	**50^a^**	**50^b^**	**100^b^**	**200^b^**	**300^b^**	**400^b^**	**300^c^**	**Total (%)**
Screened	4	4	5	5	7	2	9	36
Enrolled	4	3	3	4	4	2	6	26 (100)
Completed^d^	3	3	3	3	3	0	6	21 (80.8)
								
*Not completed*	1	0	0	1	1	2	0	5 (19.2)
Owing to pre-treatment event or adverse event	1	0	0	0	1	2	0	4 (15.4)
Owing to lack of efficacy	0	0	0	1	0	0	0	1 (3.8)
								
*Sex*								
Male	3	2	1	3	3	1	4	17 (65.4)
Female	1	1	2	1	1	1	2	9 (34.6)
								
*Age (years)*								
Median	67.0	58.0	65.0	61.0	62.5	63.0	59.5	61.5
								
*Primary tumour*								
Colon/rectal	2	3	2	3	0	2	0	12 (46.2)
Gastric	1	0	0	1	0	0	2	4 (15.4)
Oesophageal	0	0	0	0	1	0	2	3 (11.5)
Other	1	0	1	0	3	0	2	7 (26.9)
								
*Performance status*								
0	1	2	2	3	2	2	4	16 (61.5)
1	3	1	1	1	2	0	2	10 (38.5)

Abbreviations: b.i.d.=twice daily; q.d.=once daily.

a Q.d. (dose escalation cohort).

b B.i.d. (dose escalation cohort).

c B.i.d. (repeated administration cohort).

d Treatment for cycle 1 or the first 28 days completed.

**Table 2:** Frequently reported adverse events (overall incidence ⩾10%)

	**Dose level of TAK-285 (mg)**							
	**50^a^**	**50^b^**	**100^b^**	**200^b^**	**300^b^**	**400^b^**	**300^c^**	**Total (%)**
*Any adverse events*	4	3	3	4	4	2	6	26 (100.0)
⩾Grade 3	3	0	1	1	1	2	4	12
								
*Alanine aminotransferase increased*	3	0	0	2	1	2	4	12 (46.2)
⩾Grade 3	1	0	0	0	0	1	2	4
								
*Aspartate aminotransferase increased*	2	0	1	2	0	2	5	12 (46.2)
⩾Grade 3	1	0	0	0	0	1	1	3
								
*Rash*	0	1	1	2	1	2	4	11 (42.3)
⩾Grade 3	0	0	0	0	0	0	0	0
								
*Blood bilirubin increased*	2	0	1	2	0	2	2	9 (34.6)
⩾Grade 3	1	0	0	0	0	0	0	1
								
*Diarrhoea*	0	0	1	1	2	2	3	9 (34.6)
⩾Grade 3	0	0	0	0	0	0	0	0
								
*Constipation*	1	1	1	1	1	1	1	7 (26.9)
⩾Grade 3	0	0	0	0	0	0	0	0
								
*Gamma-glutamyltransferase increased*	2	0	1	2	0	1	1	7 (26.9)
⩾Grade 3	2	0	0	1	0	0	1	4
								
*Decreased appetite*	1	1	0	1	0	2	1	6 (23.1)
⩾Grade 3	1	0	0	0	0	1	0	2
								
*Pyrexia*	0	0	2	0	3	0	1	6 (23.1)
⩾Grade 3	0	0	0	0	0	0	0	0
								
*Blood alkaline phosphatase increased*	1	0	1	1	1	1	0	5 (19.2)
⩾Grade 3	0	0	0	0	0	0	0	0
								
*Blood lactate dehydrogenase increased*	1	1	1	2	0	0	0	5 (19.2)
⩾Grade 3	1	0	0	0	0	0	0	1
								
*Cancer pain*	1	1	1	0	0	0	2	5 (19.2)
⩾Grade 3	0	0	0	0	0	0	1	1
								
*Nausea*	0	1	1	0	0	2	1	5 (19.2)
⩾Grade 3	0	0	0	0	0	0	0	0
								
*Dry skin*	1	0	0	0	1	1	1	4 (15.4)
⩾Grade 3	0	0	0	0	0	0	0	0
								
*Vomiting*	0	1	1	0	0	1	1	4 (15.4)
⩾Grade 3	0	0	0	0	0	0	0	0
								
*Blood creatinine increased*	1	0	0	0	1	0	1	3 (11.5)
⩾Grade 3	0	0	0	0	0	0	0	0
								
*Haemoglobin decreased*	1	0	2	0	0	0	0	3 (11.5)
⩾Grade 3	1	0	0	0	0	0	0	1
								
*Stomatitis*	0	0	0	2	0	0	1	3 (11.5)
⩾Grade 3	0	0	0	0	0	0	0	0

Abbreviations: b.i.d.=twice daily; q.d.=once daily.

a Q.d. (dose escalation cohort).

b B.i.d. (dose escalation cohort).

c B.i.d. (repeated administration cohort).

**Table 3:** Pharmacokinetic parameters of TAK-285 after single and multiple oral dosing of TAK-285

	**Dose level of TAK-285**					
**Parameters**	**50 mg q.d.^a^**	**50 mg b.i.d.^a^**	**100 mg b.i.d.^a^**	**200 mg b.i.d.^a^**	**300 mg b.i.d.^a^**	**300 mg b.i.d.^b^**
*Single dosing*						
*N*	4	3	3	4	4	5–6
*C*_max_ (ng ml^–1^)	214 (30.3)	234 (55.6)	379 (91.8)	965 (194)	1350 (469)	983 (545)
AUC_0–∞_ (ng × h ml^–1^)	1730 (423)	1970 (648)	2820 (1190)	9530 (4150)	11 500 (4750)	9210 (3840)
*T*_max_ (h)	1.49 (1.00–2.00)	1.00 (1.00–2.02)	2.00 (1.00–4.00)	2.48 (2.00–4.00)	2.00 (2.00–2.00)	2.03 (2.00–4.00)
*t*_1/2_ (h)	7.27 (2.58)	6.45 (0.642)	9.43 (4.85)	6.57 (1.16)	7.50 (0.968)	6.10 (2.21)
						
*Multiple dosing* c						
*N*	3	3	3	3	3	5–6
*C*_max_ (ng ml^–1^)	260 (57.9)	358 (51.1)	774 (344)	2420 (156)	3700 (680)	2810 (1120)
AUC_0–tau_ (ng × h ml^–1^)	2210 (973)	2720 (778)	6090 (3140)	1700 (3970)	30 900 (6750)	26 400 (9840)
*T*_max_ (h)	2.00 (2.00–2.02)	2.05 (2.00–2.05)	2.00 (2.00–3.00)	2.00 (1.00–2.05)	2.00 (2.00–2.02)	2.97 (0.500–6.00)
*t*_1/2_ (h)	8.69 (2.87)	7.49 (0.738)	7.79 (0.760)	6.95 (1.21)	8.25 (1.61)	11.1 (3.65)
R (*C*_max_)	1.20 (0.342)	1.55 (0.205)	2.00 (0.613)	2.70 (0.829)	3.12 (1.22)	3.62 (2.05)
R (AUC)	1.35 (0.331)	1.96 (0.148)	2.79 (0.792)	3.22 (0.677)	3.92 (1.69)	4.59 (2.14)

Abbreviations: AUC=area under the plasma concentration-time curve from time zero to infinity (_0–∞_) or to dosing interval (_0–tau_); b.i.d.= twice daily; *C*_max_=maximal observed plasma concentration after dosing; q.d.= once daily; R=accumulation ratio of *C*_max_ or AUC between multiple versus single dosing; R (AUC)=AUC_0−tau_ (treatment day D)/AUC_0−12_ (treatment day 1) (dose escalation cohort: D=21, repeated administration cohort: D=28); *T*_max_=time to reach the maximal plasma concentration; *t*_1/2_=estimated terminal-phase half-life.

All parameters are reported as mean (±s.d.) values, except for *T*_max_ that is reported as a median (range) value.

a Dose escalation cohort.

b Repeated administration cohort.

c Day 21 or day 28.

**Table 4:** Pharmacokinetic parameters of M-I after single and multiple oral dosing of TAK-285

	**Dose level of TAK-285**					
**Parameters**	**50 mg q.d.^a^**	**50 mg b.i.d.^a^**	**100 mg b.i.d.^a^**	**200 mg b.i.d.^a^**	**300 mg b.i.d.^a^**	**300 mg b.i.d.^b^**
*Single dosing*						
*N*	4	3	3	4	4	6
*C*_max_ (ng ml^–1^)	57.2 (11.7)	62.6 (16.4)	165 (35.4)	313 (106)	383 (79.9)	272 (190)
AUC_0–∞_ (ng × h ml^–1^)	401 (79.3)	494 (174)	1340 (350)	2870 (242)	3230 (911)	2710 (1690)
*T*_max_ (h)	1.49 (1.00–2.00)	2.00 (1.00–2.02)	2.00 (2.00–4.00)	2.00 (2.00–4.05)	2.00 (2.00–2.00)	2.50 (2.00–3.00)
*t*_1/2_ (h)	8.32 (2.83)	7.54 (1.21)	9.70 (4.51)	7.40 (1.45)	10.0 (1.96)	6.99 (2.53)
						
*Multiple dosing* c						
*N*	3	3	3	3	3	6
*C*_max_ (ng ml^–1^)	68.0 (12.4)	96.1 (53.2)	302 (62.9)	767 (258)	549 (123)	584 (349)
AUC_0–tau_ (ng × h ml^–1^)	523 (26.3)	786 (451)	2660 (902)	6410 (3330)	4750 (1020)	5600 (3480)
*T*_max_ (h)	2.00 (1.00–2.02)	2.05 (2.00–3.00)	3.00 (3.00–4.05)	2.05 (2.00–3.00)	2.00 (2.00–2.02)	2.99 (0.500–3.98)
*t*_1/2_ (h)	11.8 (3.99)	9.57 (2.11)	10.0 (2.38)	8.53 (1.42)	14.0 (3.17)	14.3 (7.44)
R (*C*_max_)	1.19 (0.202)	1.49 (0.526)	1.90 (0.651)	2.73 (2.02)	1.58 (0.456)	2.64 (1.41)
R (AUC)	1.40 (0.216)	2.27 (0.896)	2.99 (0.740)	3.58 (2.23)	2.42 (0.859)	3.68 (1.99)

Abbreviations: AUC=area under the plasma concentration-time curve from time zero to infinity (_0–∞_) or to dosing interval (_0–tau_); b.i.d.= twice daily; *C*_max_=maximal observed plasma concentration after dosing; q.d.= once daily; R=accumulation ratio of *C*_max_ or AUC between multiple versus single dosing; R (AUC)=AUC_0–tau_ (treatment day D)/AUC_0–12_ (treatment day 1) (dose escalation cohort: D=21, repeated administration cohort: D=28); *T*_max_=time to reach the maximal plasma concentration; *t*_1/2_=estimated terminal-phase half-life.

All parameters are reported as mean (±s.d.) values, except for *T*_max_ that is reported as a median (range) value.

a Dose escalation cohort.

b Repeated administration cohort.

c Day 21 or day 28.
